# Probiotic supplementation in sustainable sheep production: impacts on health, performance, and methane mitigation

**DOI:** 10.1007/s11250-025-04439-y

**Published:** 2025-05-05

**Authors:** Ali S. A. Saleem, Sameh Abdelnour, Sabry M. Bassiony, Usama M. Abdel-Monem, Mohamed Y. Elaref, Khaled M. Al-Marakby

**Affiliations:** 1 Animal Production Department, Faculty of Agriculture, Sohag University, Sohag, Egypt; 2 Animal Production Department, Faculty of Agriculture, Zagazig University, Zagazig, Egypt

**Keywords:** Probiotics, Sheep, Environmental impacts, Health

## Abstract

Probiotics, defined as live microorganisms conferring health benefits, are increasingly recognized for their potential to enhance animal productivity, mitigate environmental impact, and improve overall animal health. Ruminants, including sheep, are significant contributors to greenhouse gas emissions, a key factor in climate change. Literature from 2003 to 2024 was retrieved from PubMed (Medline), Web of Science, and CAB Direct using the keywords: sheep, sustainability, probiotics, methane emission, and greenhouse gas emissions. The inclusion of probiotics in sheep diets demonstrates potential as a methane mitigation strategy through the stimulation of beneficial bacteria and the suppression of methanogenic microbial activity. Probiotics can improve rumen fermentation parameters by increasing volatile fatty acid production, decreasing protozoal numbers, and improving gas production. Additionally, probiotics can sustain intestinal health, boost nutrient digestibility, and strengthen the immune system. Although promising, the variable effectiveness of probiotics underscores the importance of refining formulations and delivery methods, taking into account strain, dose, and administration. Further studies are crucial to understand the underlying mechanisms and maximize their impact on sheep productivity. This review delves into the potential of probiotics to improve growth, health, and environmental sustainability in the sheep industry, drawing on insights from in vitro and in vivo studies.

## Introduction

The sheep industry is a rapidly growing sector that plays a vital role in the economic livelihoods of many developing countries. Sheep provide valuable resources, including milk and meat, while also contributing to soil health. However, sheep farming faces significant challenges, including rising feed costs and increasing competition for grain crops between humans and livestock, which adversely impacts animal growth and performance. Additionally, global warming and climate change are causing serious environmental fluctuations. Enteric fermentation in the digestive systems of ruminant animals and livestock manure accounts for significant portions of greenhouse gas emissions, contributing 39% and 20%, respectively, to the total emissions from the livestock sector (Munawaroh and Widiawati 2017; FAO 2021).

Methane, a potent greenhouse gas, contributes to global warming and energy loss in ruminants. It is produced during enteric fermentation and is a significant source of energy loss, affecting animal performance (Mar et al. 2022). Given its impact on global warming and animal energy utilization, methane has received considerable attention, leading to the implementation of various strategies to mitigate emergencies from enteric methane emissions in ruminants. Among these strategies, feed management approaches are considered the most developed methods for reducing methane emissions. However, probiotics, as a direct-fed microbial strategy, are beneficially used in animal nutrition to improve rumen fermentation, increase feed efficiency, balance the rumen microflora, and inhibit the adherence and growth of pathogens (Wang et al. 2016). Similarly, probiotics are considered a promising approach for methane mitigation (Antonius et al. 2015). It has been hypothesized that probiotics can stimulate ruminal bacterial growth and increase the bacterial population by providing them with nutrients, including metabolic intermediates and vitamins (Haque 2018). Another theory suggests that probiotics can stimulate lactic acid-utilizing bacteria, leading to a reduction in lactic acid production and the subsequent stimulation of cellulolytic bacteria growth, thereby improving fiber digestion (Mehdi 2018). Moreover, probiotics can inhibit specific rumen bacteria that produce H_2_ or methyl-containing compounds, resulting in a reduction in CH_4_ production (Doyle et al. 2019). This review focuses on the use of probiotics administration to enhance sheep health and production, while also addressing their functional roles in mitigating methane emissions and promoting sustainability in sheep farming.

### Research strategy

For this review, we conducted a comprehensive search of scientific literature using English-language sources only. Databases searched included Google Scholar, ResearchGate, PubMed, Elsevier, and Springer. The search was limited to publications from 2003 to 2024. Keywords used for article discovery included"sheep,""probiotics,""methane emission,""blood health,""sustainability,""blood biochemicals,"and"rumen fermentation."

### Probiotics

Numerous definitions for the term"Probiotics"were suggested throughout the beneficial outcomes attained from using several microbial strains in various host species (Reuben et al. 2022). According to the Food and Agricultural Organization of the United Nations (FAO 2016), the FAO and World Health Organization (WHO) definition of probiotics as “live micro-organisms that, when administered in adequate amounts, confer a health benefit on the host” is the most widely accepted.

Probiotics primarily consist of lactic acid-producing bacteria, including Lactobacilli (e.g., *Lactobacillus acidophilus*, *L. casei*, *L. lactis*, *L. rhamnosus*, *L. salivarius*) and Bifidobacteria (e.g., *Bifidobacterium longum*, *B. infantis*, *B. bifidum*), as well as yeasts like *Saccharomyces boulardii* and *S. cerevisiae* (Kumar 2013). Likewise, *Lactobacilli* and *Bifidobacterium*, *Enterococcus faecium*, and spore-forming *Bacillus spp*. are bacterial genera largely used as probiotics as well as certain yeasts such as *Saccharomyces* (Pandey et al. 2019).

### Mode of action for probiotics

Many researchers (Brown and Valiere 2004; Anandharaj et al. 2015; Hamasalim 2015) revealed that probiotics may play a beneficial role in several health conditions and performance, including intestinal microbial composition, therapeutic and metabolic effects, and immunomodulation.

Researchers have paid more attention to probiotics in the last 20 years yet a lot still remains to be uncovered (Pandey et al. 2019). Probiotics benefit the host by producing antibacterial compounds against pathogens or minimizing their competition for nutrients, changing the microbial metabolism pattern in the gut, stimulating immunity, and neutralizing pathogenic enterotoxins. In the same way, probiotics face microbial pathogens by three different mechanisms of action. These mechanisms comprise enhancing the function of the epithelial barrier, immunomodulation, and antimicrobial activity (Cerdó et al. 2019). Also, probiotics can improve the barrier function by mucin secretion, maintaining the cytoskeletal and phosphorylation of the tight junction protein, restoring the chloride secretion, and augmenting the resistance of the trans-epithelial (O′ Hara and Shanahan 2007). Moreover, probiotics could initiate the repair of the barrier function after damage. Probiotics may exert immunomodulatory effects through antimicrobial mechanisms, such as reducing luminal pH, inhibiting bacterial adherence and translocation, and secreting antibacterial substances and defensins (Ng et al. 2009). Additionally, probiotics can compete with pathogenic bacteria for binding sites to epithelial cells and the overlying mucus layer. The probiotic bacteria exert their immunomodulatory influence by interacting with epithelial and dendritic cells and with monocytes/macrophages and lymphocytes (Bermudez-Brito et al. 2012; Gogineni et al. 2013).

Probiotic administration resulted in decreased levels of inflammatory markers, including C-reactive protein and tumor necrosis factor-α, while interleukin- 6 levels remained unchanged (Naseri et al. 2023). Furthermore, a significant reduction in malondialdehyde (MDA), a marker of oxidative stress, and a concomitant increase in total antioxidant capacity, glutathione peroxidase (GSH-Px) activity, and nitric oxide levels were observed following probiotic treatment (Naseri et al. 2023).

**Fig. 1:**
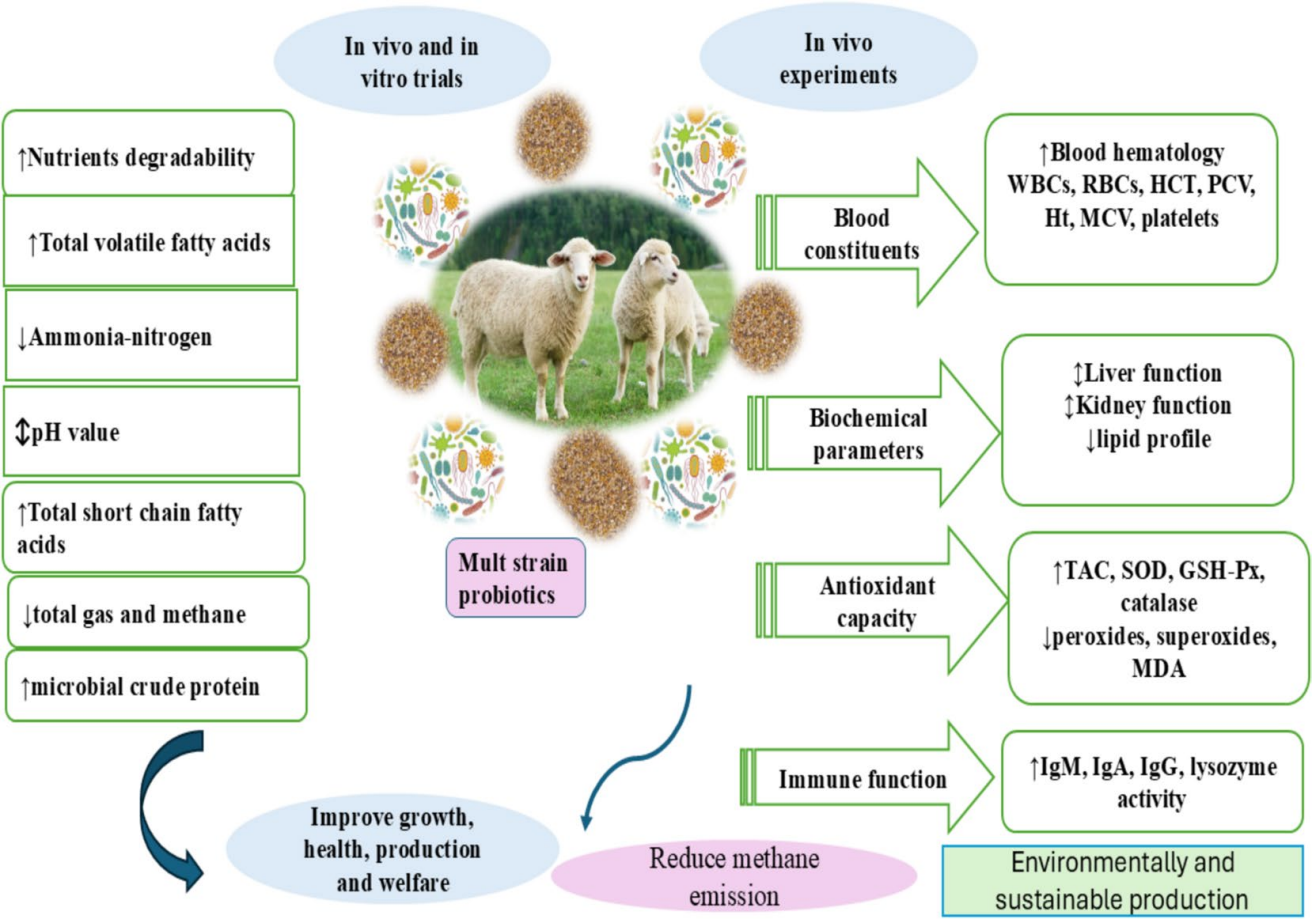
The beneficial effects of multi-strain probiotics on sheep production according to the in vivo and in vitro trials

Furthermore, in ruminants, probiotics improve rumen fermentation, and increase nutrient availability, digestibility, and utilization. The potential of the multi-species probiotic combinations to increase growth performance may be due to the improvement in fiber-degrading bacterial populations, microbial protein synthesis, and nutrient digestibility (Kulkarni et al. 2022). The role of probiotics supplementation in ruminant well-being was summarized as shown in Fig. 1.

### Effects of probiotics on in vitro nutrients degradability

The impact of incubating a dose (3 g/Kg DM) of probiotic mix (1:1) containing *Saccharomyces cerevisiae* 2 × 10^10^ CFU/g and *Lactobacillus acidophilus* 6 × 10^9^ CFU/g with feed (rice straw and concentrate mixture; 50:50) on in vitro nutrients degradability were tested by Sheikh et al. (2017). Authors detected that probiotic supplement resulted in significant improvements (*P < *0.05) of the in vitro dry matter degradability (IVDMD), in vitro organic matter degradability (IVOMD), and in vitro neutral detergent fiber degradability (IVNDFD) after 48 h of incubation. In the same trend, an in vitro fermentation of the basal diet [Alfalfa(56.2%), corn(26.4%),Soybean meal (8.4%), Wheat barn (73%), Ca_2_HPO_4_ (0.7%) and mixture of vitamins and minerals (1%)] with the live *Clostridium butyricum* (1.0 × 10^8^ CFU/g) at five doses (0, 0.025, 0.05, 0.10, and 0.20% of the dietary DM) was studied by Cai et al. (2021). Authors found that the IVDMD, IVNDFD, and acid detergent fiber degradability (IVADFD) were ameliorated (*P < *0.05) at the probiotic levels of 0.05 and 0.10% compared to the control group. Additionally, in vitro analysis revealed that sugarcane bagasse incubated with *Lactobacillus casei* TH14 at levels of 0 and 0.05 g/kg fresh matter, along with cellulase and molasses, resulted in significantly greater (*P < *0.05) in vitro IVDMD and in vitro IVNDFD compared to rice straw (So et al. 2022). Likewise, Marlida et al. (2023) fermented the rice straw-based diet with two levels (0.5 and 1%) of a probiotic mix containing *Lactobacillus plantarum *and *Saccharomyces cerevisiae* (10^10^ CFU/mL) with (60:40) for 48 h. Authors reported that the supplemental probiotics significantly enhanced (*P < *0.05) the in vitro nutrient digestibility. The addition level of 1% generated the highest increases in the IVNDFD (58.39%), IVADFD (53.99%), and cellulose (67.12%) compared to the control. From another trend, the impact of fermenting maize stover and rice straw with graded levels (0, 0.25, 0.50, and 0.75 × 10^7^ CFU/mL) of *Lactobacillus acidophilus* on the in vitro nutrients’ degradability were investigated by Chen et al. (2017), who reported that the probiotic supplementation did not have any effects on the IVDMD and IVNDFD. Table 1 presents a literature-based overview of the impacts of multi-species probiotics on in vitro of nutrients degradability sheep production parameters.

**Table 1:** A literature review of the impact of multi-species probiotics on nutrients degradability of sheep

Type of probiotics	Dose	Type of Feed Incubated	Main findings	Reference
*Saccharomyces cerevisiae, Lactobacillus acidophilus*	3 g/Kg DM of probiotic mix (1:1) containing *Saccharomyces cerevisiae* 2 × 10^10^ CFU/g and *Lactobacillus acidophilus* 6 × 10^9^ CFU/g	Rice straw + concentrate (50:50)	Increased dry matter degradability (IVDMD), organic matter degradability (IVOMD), and neutral detergent fiber degradability (IVNDFD) after 48 h of incubation	Sheikh et al. (2017)
*Clostridium butyricum*	1.0 × 10^8^ CFU/g at five doses (0, 0.025, 0.05, 0.10, and 0.20% of the dietary DM)	Basal diet	Improved (*P < *0.05) IVDMD, IVNDFD and, in vitro acid detergent fiber degradability (IVADFD)	Cai et al. (2021)
*Lactobacillus casei* TH14	0.05 g/kg fresh matter	Sugarcane bagasse	Enhanced (*P < *0.05) IVDMD), and IVNDFD	So et al. (2022)
*Lactobacillus plantarum, Saccharomyces cerevisiae*	two levels (0.5 and 1%) of a probiotic mix containing *Lactobacillus plantarum* and *Saccharomyces cerevisiae* (10^10^ CFU/mL)	Rice straw (60:40)	Increased (*P < *0.05) IVNDFD, acid detergent fiber degradability (IVADFD), and cellulose digestibility	Marlida et al. (2023)
*Lactobacillus acidophilus*	Four levels (0, 0.25, 0.50, and 0.75 × 10^7^ CFU/mL)	Maize stover + rice straw	No significant effect on dry matter degradability (IVDMD), and neutral detergent fiber degradability (IVNDFD)	Chen et al. (2017)

### Effects of probiotics on methane and total in vitro gas production

Total gas production (TGP) is affected by the microbial protein synthesis, type, and chemical composition of feed and the proportions of the volatile fatty acids (VFA) produced during fermentation (Krishnamoorthy et al. 1991). The acetate and butyrate are the primary gas production sources during fermentation (Janssen 2010; Van Lingen et al. 2016). Also, a solid relationship between the total volatile fatty acid (TVFA) production and TGP was detected when fermented feeds led to high acetate content (Rahman et al. 2013). Table 2 presents a literature-derived overview of the in vitro effects of multi-species probiotics on TGP and methane emissions associated with sheep production parameters.In an in vitro study, Wang et al. (2016) evaluated the impact of graded levels (0, 0.25, 0.50, and 0.75 × 10⁷ CFU) of *Bacillus licheniformis* and *Bacillus subtilis* on gas production kinetics and methane (CH₄) production from maize stover and rice straw. The authors found that *Bacillus licheniformis* and *Bacillus subtilis* significantly increased total gas production (TGP; *P < *0.05). Compared to the control, *Bacillus licheniformis* reduced methane (CH₄) production, and *Bacillus subtilis* increased the acetate-to-propionate ratio. In a separate experiment, the methanogenic reduction potential of lactic acid bacteria (LAB), bifidobacteria, and propionibacteria were determined through a 24-h incubation in vitro. Some probiotics contain enzymes like beta-galactosidase, which can help break down lactose (Cai et al. 2021). This is particularly beneficial for individuals with lactose intolerance, as it reduces the fermentation of lactose and subsequent gas production. Three strains (*Propionibacterium freudenreichii* 53-W at 6 × 10^10^ CFU/day, *Lactobacillus pentosus* D31 at 6 × 10^10^ CFU/day, and *Lactobacillus bulgaricus* D1 at 3 × 10^10^ CFU/day) had the greatest impact on TGP and CH_4_ production (Jeyanathan et al. 2016). In addition, all treatments significantly increased the TGP and reduced the CH_4_ production (*P < *0.05) compared to the control (Jeyanathan et al. 2016). Also, the effects of four levels (0, 0.25, 0.50, and 0.75 × 10^7^ CFU/mL) of *Lactobacillus acidophilus* supplementation were examined on CH_4_ production and gas production of maize stover and rice straw (Chen et al. 2017). Authors found that the supplementation level of *Lactobacillus acidophilus* did not affect CH_4_ production. Authors added that *Lactobacillus acidophilus* increased the in vitro gas production when using either fermentation substrate, with the greatest increase observed at the highest tested level of (0.75 × 10^7^ CFU/mL). Likewise, sorghum silage samples were fermented with *Lactobacillus* bacteria using an in vitro gas production technique (Khota et al. 2017). Authors indicated that all probiotics significantly increased (*P < *0.05) the TGP and reduced the CH_4_ production compared to control. The ratio of CH_4_ production to gas production was significantly (*P < *0.05) reduced with *Lactobacillus casei* than *Lactobacillus plantarum*. The effects of bacterial probiotics (*Propionibacterium* spp., *Propionibacterium* + *Lactobacillus plantarum*, and *Propionibacterium* + *Lactobacillus plantarum* 115 + *Lactobacillus rhamnosus* 32) administered at 10^10^ CFU/day compared to the control (without the probiotics) were evaluated on ruminal fermentation and CH_4_ emission in dairy cows by Philippeau et al. (2017). The CH_4_ emission decreased in cows fed a low-starch diet supplemented with a combination of *Propionibacterium* + *Lactobacillus plantarum*. Nonetheless, they observed that the probiotic supplements did not significantly alter ruminal volatile fatty acid (VFA) concentrations. In vitro experiments with 14 *Lactobacillus plantarum* strains showed that strain U32 had the lowest relative CH₄ production (Astuti et al. 2018). The authors found that the gas fraction increase was positively correlated with TGP, the rate of gas production, and the maximum volume of gas produced. Similarly, Abdelbagi et al. (2021) investigated the effects of probiotics (10 LAB strains) on in vitro TGP and CH_4_ production. The probiotics were administered in either liquid form (0.5 mL; 10^11^ CFU/mL) or encapsulated form (0.5 g freeze-dried; 10^11^ CFU/g) and compared to a control with no additives. Diets (40:60 forages to concentrate) samples were incubated for 72 h. Authors revealed that the liquid and freeze-dried probiotics decreased (*P < *0.05) CH_4_ production by 6.1 and 33.1%, respectively compared to the control diet. Additionally, both probiotics forms elevated (*P < *0.05) the TGP (mg/g DM). Sugarcane bagasse was fermented with *Lactobacillus casei* TH14 (at 0 and 0.05 g/kg fresh matter), cellulase, and molasses, and the results were compared to those obtained with rice straw (So et al. 2022). Authors reported that the sugarcane bagasse fermented with *Lactobacillus* casei TH14, cellulase, and molasses had greater (*P < *0.05) gas from soluble fraction and rate constant of gas production. Lastly, the study evaluated the effects of two probiotic formulations, *Ligilactobacillus animalis* and *Propionibacterium freudenreichii* (1.5 × 10⁷ CFU/mL) or *Bacillus subtilis* and *B. licheniformis* (5.9 × 10⁷ CFU/mL), on TGP and CH₄ production during the in vitro fermentation of maize silage and grass silage was mentioned by Dhakal et al. (2023). For 48 h, freeze-dried samples of both silages and buffer were incubated in rumen fluid and with *Ligilactobacillus animalis* plus *Propionibacterium freudenreichii* (1.5 × 10^7^ CFU/mL), and *Bacillus subtilis* and *Bacillus licheniformis* (5.9 × 10^7^ CFU/mL). The maize silage produced significantly more TGP than grass silage after 48 h. However, grass silage with probiotics produced significantly more gas at 3 and 9 h and a similar gas volume at 12 h. Both probiotics increased the TGP significantly in grass silage at 48 h. Adding a mix of* Bacillus subtilis* and *Bacillus licheniformis* (5.9 × 10^7^ CFU/mL) reduced the total CH_4_ production by 4–6% in feeds.

**Table 2:** A literature review of the impact of multi-species probiotics on methane and total in vitro gas production of sheep (in vitro)

Type of probiotics	Dose	Type of Feed Incubated	Main findings	Reference
*Bacillus licheniformis, Bacillus subtilis*	0, 0.25, 0.50, and 0.75 × 10^7^ CFU	Maize stover + rice straw	Increased (*P* < 0.05) total gas production (TGP), reduced methane (CH_4_)	Wang et al. (2016)
*Propionibacterium freudenreichii 53*-W, *Lactobacillus pentosus* D31,* Lactobacillus bulgaricus* D1	*Propionibacterium freudenreichii* 53-W at 6 × 10^10^ CFU/day, *Lactobacillus pentosus* D31 at 6 × 10^10^ CFU/day, and *Lactobacillus bulgaricus* D1 at 3 × 10^10^ CFU/day)	Sheep diet	All treatments significantly increased the total gas production, while *Propionibacterium freudenreichii* 53-W Increased CH_4_ (16%), *Lactobacillus pentosus* D31 reduced CH_4_ (13%), *Lactobacillus bulgaricus *did not affect CH_4_ production	Jeyanathan et al. (2016)
*Lactobacillus acidophilus*	Four levels (0, 0.25, 0.50, and 0.75 × 10^7^ CFU/mL)	Maize stover + rice straw	Increased the total gas production, no effect on CH_4_ production	Chen et al. (2017)
*Lactobacillus casei* TH14, *Lactobacillus plantarum*	*Lactobacillus casei* TH14 (5.3 × 10^7^) *Lactobacillus plantarum*(1.5 × 10^5^)	Sorghum silage	Increased (*P* < 0.05) the total gas production and reduced the CH_4_ production	Khota et al. (2017)
*Propionibacterium spp., Lactobacillus plantarum, Lactobacillus rhamnosus 32*	10^10^ CFU/day	a high-starch diet (HS; 38%) or a low-starch diet (LS; 2%) in a 55:45 forage:concentrate ratio	Reduced CH4 in low-starch diet	Philippeau et al. (2017)
*Lactobacillus plantarum strain* U32	Tested *in vitro*	Various feeds	Highest TGP, Lowest CH_4_ production among tested strains	Astuti et al. (2018)
*Lactobacillus casei* TH14	0.05 g/kg fresh matter	Sugarcane bagasse	Increased TGP, reduced CH_4_	So et al. (2022)
*Ligilactobacillus animalis, Propionibacterium freudenreichii*	1.5 × 10^7^ CFU/mL	Maize silage, grass silage	Increased TGP, reduced CH_4_ production	Dhakal et al. (2023)
*Bacillus subtilis, Bacillus licheniformis*	5.9 × 10^7^ CFU/mL	Maize silage, grass silage	Reduced CH_4_ production by 4–6%	Dhakal et al. (2023)
*10 LAB strains (liquid and freeze-dried forms)*	Liquid form (0.5 mL; 10^11^ CFU/mL) or encapsulated form (0.5 g freeze-dried; 10^11^ CFU/g)	Mixed forages (40:60)	Reduced CH_4_ production by 6.1% (liquid) and 33.1% (freeze-dried)	Abdelbagi et al. (2021)

### Effect of probiotics on fermentation parameters and protozoa counts

#### Total volatile fatty acids

Volatile fatty acids (VFAs), produced by rumen fermentation, significantly impact ruminant production and product composition. This is largely due to their role as the primary energy source for ruminants, providing approximately 75% of their metabolizable energy. Paengkoum et al. (2011) investigated a probiotic combination of *Saccharomyces cerevisiae* and *Lactobacillus acidophilus* at a dose of 10^1^⁰ CFU/g dry matter (DM). This combination increased average total volatile fatty acid (TVFA) concentration, with a greater effect observed at higher daily probiotic doses. A separate study examined a 3 g/kg DM dose of a 1:1 probiotic blend consisting of *Saccharomyces cerevisiae* (2 × 10^1^⁰ CFU/g) and *Lactobacillus acidophilus* (6 × 10⁹ CFU/g) added to a 50:50 rice straw and concentrate feed mixture. After 48 h of incubation, the TVFA concentration in rumen fluid was significantly (*P < *0.01) elevated compared to the control (Sheikh et al. 2017). Furthermore, a dose of LAB (10 strains) as a solution (0.5 mL; 10^11^ CFU/mL) or encapsulated (0.5 g of freeze-dried; 10^11^ CFU/g) compared to control (no additives) was tested on the in vitro production of TVFA (for 72 h). Another study has proved that probiotics significantly improved the TVFA compared to control (Abdelbagi et al. 2021). The effects of different roughages in total mixed ration inoculated with or without *Lactobacillus acidophilus* and *Bacillus subtilis* were determined on in vitro fermentation (Miguel et al. 2021). After 24 h of incubation, authors found that the TVFA concentrations were higher (*P < *0.05) than that in the control. In the same line, Marlida et al. (2023) supplemented the rice straw-based rations (60:40) with probiotics containing *Lactobacillus plantarum *and *S. cerevisiae* (10^10^ CFU/mL) on in vitro ruminal characteristics. Probiotic treatment significantly increased (*P < *0.05) TVFA level by 11% compared to the control. Chen et al. (2017) evaluated the effects of graded *Lactobacillus acidophilus* doses (0, 0.25, 0.50, and 0.75 × 10⁷ CFU/mL) on the in vitro fermentation of maize stover and rice straw. Authors found that none of the tested probiotic levels affected TVFA concentration. Likewise, the effect of two probiotics [*Ligilactobacillus animalis* plus *Propionibacterium freudenreichii* (1.5 × 10^7^ CFU/mL), and *Bacillus subtilis* and *Bacillus licheniformis* (5.9 × 10^7^ CFU/mL)] formulations were evaluated on rumen fermentation with maize silage and grass silage, and results indicated that no differences in TVFA concentration were noted (Dhakal et al. 2023). Based on literature, probiotics enhance VFA production through two key mechanisms: increased carbohydrate breakdown, resulting in more VFA byproducts, and a shift in the gut's microbial composition towards VFA-producing bacteria.

### Ammonia–nitrogen

Changes in ammonia levels can have varied effects depending on the specific context in rumen physiology. Increased ammonia can benefit fibrolytic bacteria and improve fiber digestion. Conversely, decreased ammonia can reduce nitrogen waste and improve nitrogen use efficiency. The effects of *Bacillus licheniformis* and* Bacillus subtilis* were tested at graded levels (0, 0.25, 0.50, and 0.75 × 10^7^ CFU) on in vitro fermentation of maize stover and rice straw (Wang et al. 2016). Authors revealed that *Bacillus licheniformis* elevated (*P < *0.05) the NH_3_-N level, while *Bacillus subtilis* reduced (*P < *0.05), compared to the control. As well, maize stover and rice straw were fermented with graded levels (0, 0.25, 0.50, and 0.75 × 10^7^ CFU/mL) of *Lactobacillus acidophilus* to study its influences on the in vitro NH_3_-N concentration (Chen et al. 2017). Authors indicated that *Lactobacillus acidophilus* increased the NH_3_-N concentration. The most elevated NH_3_-N level was noted with the highest dose of probiotic (0.75 × 107 CFU/mL). Also, the effects of live or autoclaved *Bacillus subtilis natto* were investigated on ruminal NH_3_-N concentrations (Chang et al. 2021). Authors elucidated that the NH_3_-N level increased significantly in the autoclaved *Bacillus subtilis natto* within 24 h, while it was higher before 6 h after adding live *Bacillus subtilis natto*, with no difference after 12 h compared to the control. Moreover, the *Bacillus subtilis* significantly (*P < *0.05) increased the levels of NH_3_-N (by 7.83%) compared to the control after 24 h of fermentation. Likewise, a dose of LAB (10 strains) as a solution (0.5 mL; 10^11^ CFU/mL) or encapsulated (0.5 g of freeze-dried; 10^11^ CFU/g) compared to control (no additives) was assessed on the in vitro NH_3_-N concentration (for 72 h). The liquid form of probiotics significantly raised (*P = *0.008) the NH_3_-N concentration compared to the control and the encapsulated probiotics (Abdelbagi et al. 2021). Moreover, the effect of probiotics containing *Lactobacillus plantarum *and *Saccharomyces cerevisiae* (10^10^ CFU/mL) were examined on the in vitro ruminal characteristics. The level of NH_3_-N was enhanced (*P < *0.05) from 22.59 mg/100 mL in the control to 26.56 mg/100 mL in the probiotic treatment (Marlida et al. 2023).

On the other hand, a dose of 3 g/Kg DM of probiotic blend (1:1) comprised of *Saccharomyces cerevisiae* 2 × 10^10^ CFU/g and *Lactobacillus acidophilus* 6 × 10^9^ CFU/g with feed (rice straw and concentrate mixture; 50:50) were incubated. There was a minor decrease in the NH_3_-N concentration after 48 h of in vitro incubation paralleled to the control (Sheikh et al. 2017). Additionally, Sızmaz et al. (2020) studied the impacts of a probiotic combination (involving* Saccharomyces cerevisiae, Bacillus subtilis, Bifidobacterium bifidum, Bifidobacterium animalis, Bifidobacterium longum, Lactobacillus acidophilus, Streptococcs thermophiles, Lactobacillus casei, Lactobacillus plantarum, Lactobacillus bulgaricus*, and *Lactobacillus fermentum*) on the in vitro NH_3_-N (for 48 h). Authors clarified that the NH_3_-N concentrations tended to reduce (*P > *0.05) with probiotic treatment. By enhancing the overall efficiency of digestion and nutrient absorption, probiotics can help animals utilize dietary nitrogen more effectively. This reduces the amount of excess nitrogen that is converted into ammonia.

### pH value

Rumen pH is a useful indicator of optimal rumen conditions, proper fermentation, nutrient digestibility, and overall rumen health (Faniyi et al. 2019; Sari et al. 2019). Probiotics can positively influence ruminant health and productivity by stabilizing the rumen environment (Kulkarni et al. 2022). The combined use of *Saccharomyces cerevisiae* with *Lactobacillus acidophilus* (10^10^ CFU/g DM) increased the average rumen pH value (Paengkoum et al. 2011). As well, the probiotic supplementation increased the pH value in the rumen with increasing levels of *Saccharomyces cerevisiae* (10^10^ CFU/g of DM) from 0.5 to 5 g/day (Pinloche et al. 2013). Also, a dose (3 g/Kg DM of probiotic blend (1:1) comprised of *Saccharomyces cerevisiae* 2 × 10^10^ CFU/g and *Lactobacillus acidophilus* 6 × 10^9^ CFU/g with feed (rice straw and concentrate mixture; 50:50) increased the pH value of the rumen fluid (*P < *0.01) by 0.54 units compared to the control (Sheikh et al. 2017). Autoclaved *Bacillus subtilis natto* increased rumen fluid pH after 6 and 12 h of in vitro incubation, while the live *Bacillus subtilis natto* increased it at 24 h compared to the control. This pH increase with *Bacillus subtilis* may be associated with higher NH_3_-N levels in the rumen fluid (Chang et al. 2021). In a separate study, (Miguel et al. 2021) observed lower rumen fluid pH values (*P < *0.05) after 24 h of in vitro fermentation with *Lactobacillus acidophilus* and *Bacillus subtilis*. The authors hypothesized that the yeast may compete with lactate-producing bacteria while also promoting lactate-utilizing bacteria, thereby reducing lactate accumulation.

Probiotics can increase rumen pH by reducing lactic acid accumulation, promoting amylolytic bacteria, and potentially increasing ammonia production (Chang et al. 2021). These mechanisms help maintain a more stable pH in the rumen by reducing the acidity levels. Probiotics can also influence the buffering capacity of the rumen by altering microbial populations and fermentation patterns (Sheikh et al. 2017; Miguel et al. 2021). Overall, probiotics play a crucial role in maintaining a healthy rumen environment by supporting beneficial microbial activity and reducing the risk of acidosis.

On the contrary, the effect of three probiotic strains (*Propionibacterium freudenreichii* 53-W at 6 × 10^10^ CFU, *Lactobacillus pentosus* D31 at 6 × 10^10^ CFU, and *Lactobacillus bulgaricus* D1 at 3 × 10^10^ CFU) were evaluated on fermentation parameters (Jeyanathan et al. 2016). Authors found that there was no effect of these probiotic supplements on the in vitro pH values of the rumen fluid. Similarly, the effects of supplementing maize stover and rice straw with *Lactobacillus acidophilus* at four ascending levels (0, 0.25, 0.50, and 0.75 × 10^7^ CFU/mL) were tested in vitro ruminal pH values (Chen et al. 2017). Authors elucidated that the probiotic did not affect the ruminal pH value. Likewise, Sızmaz et al. (2020) observed that the in vitro incubation of the probiotic combination for 48 h did not alter the pH value of the rumen fluid. Also, the effects of probiotics [LAB as a solution (0.5 mL; 10^11^ CFU/mL) or freeze-dried (0.5 g of 10^11^ CFU/g)] compared to control (no supplements) were examined on the in vitro (72 h) pH values (Abdelbagi et al. 2021). Authors demonstrated that supplementation with probiotics resulted in a small decrease in the pH value of the rumen fluid compared to the control. Furthermore, probiotic supplements comprising *Lactobacillus plantarum *and *Saccharomyces cerevisiae* (10^10^ CFU/mL) did not alter (*P > *0.05) the pH value (6.76—6.80) after 48 h of in vitro incubation (Marlida et al. 2023). Discrepancies in reports regarding probiotic-induced pH declines in the rumen can be explained by several factors, including: probiotic strain specificity, substrate availability, dosage and duration of administration, initial rumen environment, experimental design, and animal breed.

### Protozoa count

Rumen protozoa play a complex and multifaceted role within the rumen ecosystem. The effect of 3 probiotic strains (*Propionibacterium freudenreichii* 53-W at 6 × 10^10^ CFU, *Lactobacillus pentosus* D31 at 6 × 10^10^ CFU, and *Lactobacillus bulgaricus* D1 at 3 × 10^10^ CF) was evaluated on in vitro fermentation parameters (Jeyanathan et al. 2016). Authors illustrated that these probiotic strains did not affect the number of protozoa. Nonetheless, the in vitro incubation (for 48 h) of a probiotic formulation that consisted of* Saccharomyces cerevisiae, Bacillus subtilis, Bifidobacterium bifidum, Lactobacillus acidophilus, Lactobacillus casei, Lactobacillus plantarum,* and* Lactobacillus bulgaricus* increased (*P < *0.001) the total numbers of protozoa compared to that in control (Sızmaz et al. 2020). Authors added that the probiotics might modulate microbial metabolic activity and the population of ruminal microorganisms.

### Total short chain fatty acids and microbial crude protein

Short-chain fatty acids (SCFAs) and microbial crude protein (MCP) are essential for animal health, especially in digestive physiology and nutrient absorption. SCFAs support cell growth, reduce inflammation, and strengthen the gut barrier, preventing intestinal permeability and bacterial translocation. MCP is a key protein source produced by microbial fermentation of dietary carbohydrates in the rumen, enhancing nutrient availability by converting low-quality dietary protein into high-quality microbial protein.

Using *Saccharomyces cerevisiae* with *Lactobacillus acidophilus* (10^10^ CFU/g) raised the short-chain fatty acids (SCFA) with a more noticeable influence with a dose of 5 g/day (Paengkoum et al. 2011). Likewise, the supplemental probiotic increased the total concentration of SCFA in the rumen with the increasing levels of *Saccharomyces cerevisiae* (10^10^ CFU/g of DM) from 0.5 to 5 g/day (Pinloche et al. 2013). Also, the microbial crude protein (MCP) content was higher with live *Bacillus subtilis natto* (10^9^ CFU) than with the control after 12 h of in vitro fermentation with dairy rations. *Bacillus subtilis* enhanced (*P < *0.05) the MCP content by 41.46% compared to the control after 24 h of the in vitro fermentation (Chang et al. 2021). Following a 48-h in vitro incubation with a probiotic compound comprising *Saccharomyces cerevisiae*, *Bacillus subtilis*, *Bifidobacterium bifidum*, *B. animalis*, *B. longum*, *Lactobacillus acidophilus*, *Streptococcus thermophilus*, *L. casei*, *L. plantarum*, *L. bulgaricus*, and *L. fermentum*, Sızmaz et al. (2020) observed a non-significant (*P > *0.05) trend towards elevated total short-chain fatty acid (SCFA) concentrations compared to the control.

### In vivo evaluation of probiotic supplements

#### Nutrient digestibility and nutritive values

A digestibility trial using male yearling Rahmani sheep carried out to assess the impact of supplementing the basal diet (concentrate feed mixture: roughage; 60:40%) with 5 or 7.5 g of live dried yeast* Saccharomyces cerevisiae*/head/day on some rumen characteristics (Mousa et al. 2012). Authors found that the digestibility values of DM, CP, and CF were higher with the yeast-supplemented group than the control one (*P < *0.05). *Saccharomyces cerevisiae* supplementation improved the nutritive value of total digestible nutrients (TDN) and digestible crude protein (DCP). In the same manner, a dietary probiotic blend (including* Lactobacillus acidophilus, Lactobacillus casei, Bacillus licheniformis,* and others) resulted in better DM, OM, CP, CF, and EE (*P < *0.05) digestibility values than the control in Barki rams (Soliman et al. 2016). Besides, Saleem et al. (2017) tested the impacts of including two dietary levels (0.5 or 1 g/day) of bacterial probiotics (*Pediococcus spp*.) on nutrients digestibility in Saidi lambs. Authors illustrated that the digestibility values of DM, OM, CP, CF, and NFE were improved (*P < *0.05) with the probiotics compared to the control. Probiotic diets also significantly improved nutritional values, including total digestible nutrients (TDN) and digestible crude protein (DCP), compared to the control diet (Saleem et al. 2017). Also, the effects of three doses (2.5 × 10^8^, 2.5 × 10^9^, or 2.5 × 10^10^ CFU/animal/day) of *Bacillus licheniformis* in Dorper crossbred male sheep (45.0 ± 1.96 kg of body weight) diets were examined on apparent digestibility (Deng et al. 2018). Authors found that the probiotic treatments improved (*P < *0.001) the apparent digestibility of DM, OM, CP, and neutral detergent fiber (NDF), the N retention and utilization efficiency (*P < *0.005), and metabolizable energy (*P < *0.001). Furthermore, the impact of a dietary probiotic including *Bacillus subtilis* (0.0252 × 10^6^ CFU/Kg diet/day) on the apparent digestion coefficients of nutrients in buffalo calves were studied (Mousa and Marwan 2019). Authors noted that the probiotic supplementation improved all nutrient digestibility’s (*P* ≤ 0.05) compared to the control group. In a similar way, Sallam et al. (2020) scrutinized the influences of supplementing growing Barki lambs’ diets (peanut hay and concentrates; 50:50) with at 0.5 g/day of a probiotic combination containing *Saccharomyces cerevisiae*, LAB, and exogenous enzymes on nutrient digestibility. Authors documented that the probiotic supplement upgraded the digestibility of neutral detergent fiber (*P = *0.020) and acid detergent fiber (*P = *0.034) compared to the control treatment. Likewise, in a study involving Farafra lambs fed a palm leaf hay-based control diet, Hamdon et al. (2022) found that supplementation with *Bacillus subtilis* significantly enhanced (*P < *0.05) the digestibility of dry matter, organic matter, crude protein, and neutral detergent fiber. However, supplementation with *Lactobacillus acidophilus* did not significantly alter the digestibility of these parameters. *S. cerevisiae* enhances the nutritive value of TDN and DCP by improving rumen fermentation. It boosts fiber digestion by stimulating fiber-digesting bacteria, leading to better breakdown of complex carbohydrates and increased nutrient availability (Deng et al. 2018; Sallam et al. 2020). This yeast also enhances protein utilization by promoting the growth of nitrogen-efficient bacteria, increasing microbial protein synthesis. Additionally, it stabilizes rumen pH, creating a favorable environment for beneficial microbes and improving nutrient digestibility (Saleem et al. 2017). The overall result is increased TDN and DCP due to a more efficient digestive process. Table 3 presents a literature-based summary of the effects of probiotics on in vivo nutrient digestibility and nutritive values in sheep.

**Table 3:** Summary of the effects of probiotics on in vivo nutrient digestibility and nutritive values in sheep based on literature

Type of probiotics	Dose/Level	Sheep breed	Type of Feed	Main findings	Reference
*Saccharomyces cerevisiae*	5–7.5 g/head/day	Rahmani sheep	Concentrate: Roughage (60:40%)	Improved dry matter (DM), crude protein (CP), crude fiber (CF), total digestible nutrients (TDN) and digestible crude protein (DCP)	Mousa et al. (2012)
*Lactobacillus acidophilus, Lactobacillus casei, Bacillus licheniformis*	Basal rations plus either 10 g probiotics (DFM)/head/day Total microbial activity, min: 1.6 billion (1.6 × 10^9^) CFU	Barki rams	60% concentrate feed mixture (CFM) and 40% clover hay	Enhanced (*P* < 0.05) DM, OM, CP, CF, EE digestibility	Soliman et al. (2016)
A mixture of two strains of *Pediococcus, Pediococcus acidilactici *and* Pediococcus pentosaceus*	A dose rate of 0, 0.5, and 1 g probiotics/lamb/day Pediococcus, Pediococcus acidilactici (1 × 10^6^ cfu/g) and Pediococcus pentosaceus (1.3 × 10^6^ cfu/g), with dextrose as the carrier compound	Saidi lambs	Concentrate and wheat straw at a concentrate: roughage ratio of 80:20	Increased DM, OM, CP, CF, NFE digestibility, total digestible nutrients (TDN) and digestible crude protein (DCP)	Saleem et al. (2017)
*Bacillus licheniformis*	2.5 × 10⁸ (low), 2.5 × 10^9^(medium), 2.5 × 10^1^⁰ (high) CFU/animal/day	Dorper crossbred male sheep	Mixed diet (34:66%; concentrate to forage)	Improved (*P* < 0.001) DM, OM, CP, NDF digestibility, metabolizable energy, and nitrogen retention (*P* < 0.005)	Deng et al. (2018)
*Bacillus subtilis*	0.0252 × 10⁶ CFU/Kg diet/day	Buffalo calves	concentrate feed mixture (CFM), wheat straw and berseem	Increased (*P* ≤ 0.05) all nutrient digestibility	Mousa and Marwan (2019)
*Saccharomyces cerevisiae*, LAB, *exogenous enzymes*	0.5 g/day	Barki lambs	Peanut hay and concentrates (50:50)	Increased NDF and ADF digestibility	Sallam et al. (2020)
*Bacillus subtilis*	4 g of Bacillofort containing 2 × 10^11^ CFU of *Bacillus subtilis*/g	Farafra growing lambs	70% concentrate + 30% date palm leaves	Increased DM, OM, CP, NDF digestibility, TDN, and DCP	Hamdon et al. (2022)
*Lactobacillus acidophilus*	4 g of Lacotpro containing 1 × 10^12^ CFU of *Lactobacillus acidophilus*/g	Farafra growing lambs	70% concentrate + 30% date palm leaves	No significant effect on digestibility of DM, CP, or NDF, TDN, and DCP compared to the control	Hamdon et al. (2022)

### Rumen fermentation

Rumen fermentation, a critical anaerobic process within the rumen, facilitates fiber digestion and the synthesis of VFAs, the primary energy source for ruminants. This microbial fermentation also enables the production of microbial protein and B vitamins. Optimizing rumen fermentation is a key indicator of nutrient digestibility in ruminants, directly influencing their growth and production performance.

A digestibility study was conducted to assess the impact of supplementing the basal diet (concentrate feed mixture: roughage; 60:40%) with 5 or 7.5 g of *Saccharomyces cerevisiae*/head/day on some rumen characteristics in Rahmani sheep by Mousa et al. (2012). Authors detected an increase in the ruminal total volatile fatty acids (TVFA) level with both doses of the yeast after three hours of feeding. In the same trend, Sheikh et al. (2022) also supplemented Corriedale sheep with *Lactobacillus acidophilus* (6 × 10⁹ CFU/g) and *Saccharomyces cerevisiae* (2 × 10^1^⁰ CFU/g) to investigate their effects on rumen fermentation (Table 4). Authors reported a significant increase (*P < *0.01) in ruminal TVFA concentration with probiotic supplementation. Moreover, the effects of adding 4 g of BAC (2 × 10^11^ CFU of *Bacillus subtilis*/g) or ZAD (6 × 10^8^ CFU of *Ruminococcus albus*/g) were studied on rumen parameters in a metabolism trial on Farafra male lambs (Hamdon et al. 2022). BAC and ZAD supplementation increased ruminal TVFA concentrations in Farafra sheep compared to controls, likely due to increased ruminal microbial activity, fermentation rate, and availability of fermentable carbohydrates. Furthermore, supplementing Holstein cows with *Bacillus licheniformis* resulted in a significant elevation (*P < *0.05) of ruminal TVFA (Qiao et al. 2010). Also, Mousa and Marwan (2019) studied the effect of a dietary probiotic containing *Bacillus subtilis* (0.0252 × 10^6^ CFU/Kg diet/day) on the rumen fermentation of buffalo calves. There was a significant increase (*P* ≤ 0.05) in TVFA compared to the control group. Probiotics increase ruminal TVFA by introducing beneficial microbes, promoting the growth of VFA-producing bacteria, improving fiber digestion and substrate utilization, and generally enhancing the efficiency of rumen fermentation (Hamdon et al. 2022; Sheikh et al. 2022).Table 4 summarizes the effects of probiotics on in vivo rumen fermentation of sheep. From another standpoint, no change in the ruminal TVFA concentration with the dietary mixture of *Saccharomyces cerevisiae* (4 × 10^9^ CFU) and *Bacillus licheniformis* (6 × 10^9^ CFU) were observed in fattening sheep (Jia et al. 2018). Moreover, Chen et al. (2021) demonstrated that probiotic supplementation with a mixture of *Bacillus licheniformis* and *Bacillus subtilis* (1:1:0.5) had no significant impact on ruminal TVFA levels in Chuanzhong black female lambs. Similarly, the impact of three probiotic formulations (*Lactobacillus fermentum* + *Lactobacillus plantarum*; *Saccharomyces cerevisiae* + *Lactobacillus fermentum* + *Lactobacillus plantarum*; and *Megasphaera elsdenii* + *Saccharomyces cerevisiae* + *Lactobacillus fermentum* + *Lactobacillus plantarum*) on ruminal fermentation in Arabian fattening lambs observed that the concentration of TVFA was not impacted (*P > *0.05) by the probiotic treatments (Direkvandi et al. 2020b).A significant decrease in ruminal NH_3_-N levels (*P < *0.05) was observed when *S. cerevisiae* was included in the diets of Rahmani sheep (Mousa et al. 2012). Also, the concentrations of NH_3_-N significantly declined (*P = *0.007), with dietary *S. cerevisiae* alone or a mixture of *S. cerevisiae* and *Bacillus licheniformis* in fattening lambs (Jia et al. 2018). In the same line, a beneficial impact of the dietary *Saccharomyces cerevisiae* was declined on the ruminal NH_3_-N concentrations in growing goats (Ogbuewu and Mbajiorgu 2023). Similarly, in Holstein cows, ammonia nitrogen (NH_3_-N) concentration significantly lessened (*P < *0.05) three hours post-feeding with *Bacillus licheniformis* supplementation (Qiao et al. 2010).

**Table 4:** Summary of in vivo rumen fermentation responses to probiotic administration in sheep

Type of probiotics	Dose/Level	Sheep breed	Type of Feed	Main findings	Reference
*Saccharomyces cerevisiae*	5–7.5 g/head/day	Rahmani sheep	Concentrate feed mixture: roughage; 60:40%	A significant increase (*P* < 0.05) in ruminal total volatile fatty acid (TVFA) concentrations and a significant decrease (P < 0.05) in ruminal ammonia nitrogen (NH_3_-N) concentrations were observed	Mousa et al. (2012)
*Lactobacillus acidophilus*, *Saccharomyces cerevisiae*	probiotics (*Saccharomyces cerevisiae* 2 × 10^10^ cfu/g + *Lactobacillus acidophilus* 6 × 10^9^ cfu/g)	Corriedale sheep	Rice straw and concentrate mixture; 50:50)	A significant increase (*P* < 0.01) in ruminal total volatile fatty acid (TVFA) concentrations was observed	Sheikh et al. (2022)
*Bacillus subtilis* (BAC), *Ruminococcus albus* (ZAD)	4 g (BAC: 2 × 10^11^ CFU/g, ZAD: 6 × 10⁸ CFU/g)	Farafra growing lambs	70% concentrate + 30% date palm leaves	A significant increase (*P* < 0.05) in ruminal TVFA concentrations was observed, along with a slight decrease in NH_3_-N concentrations	Hamdon et al. (2022)
*Saccharomyces cerevisiae, Bacillus licheniformis*	4 × 10⁹ CFU, 6 × 10⁹ CFU	Fattening sheep	Concentrate: roughage ratio of 40:60	There was no significant effect on TVFA concentrations; however, ruminal NH_3_-N concentrations showed a significant decrease (*P = *0.007)	Jia et al. (2018)
*Lactobacillus fermentum*, *Lactobacillus plantarum*, *Saccharomyces cerevisiae*, *Megasphaera elsdenii*	4.5 × 10^8^ cfu/day of L. *plantarum* and *L. fermentum* (in ratio 50:50), and *Saccharomyces cerevisiae* 1.4 × 10^10^ cfu/day	Arabian lambs	70% concentrate and 30% forage	No significant effect on the ruminal TVFA and NH_3_-N	Direkvandi et al. (2020b)
*Bacillus licheniformis, Bacillus subtilis*, and *Lactobacillus plantarum*	0.1% probiotics at a ratio of 1:1:0.5	Chuanzhong black lambs	50% concentrate and 50% forage	No significant effect on the ruminal TVFA and NH_3_-N	Chen et al. (2021)

Contrarily, the ruminal NH_3_-N level was significantly elevated (*P* ≤ 0.05) when the buffalo calves received a dietary probiotic containing *Bacillus subtilis* in comparison with the control group (Mousa and Marwan 2019). In another light, Direkvandi et al. (2020b) investigated the effects of three different probiotic combinations (using *Lactobacillus fermentum*, *Lactobacillus plantarum*, *Saccharomyces cerevisiae*, and *Megasphaera elsdenii*) on ruminal fermentation in male Arabian lambs. Authors noticed that the probiotic supplements did not impact the ruminal NH_3_-N concentrations (*P > *0.05). In the same trend, probiotics supplements (*Bacillus licheniformis* and *Bacillus subtilis*) had no substantial effect on the ruminal NH_3_-N levels in Chuanzhong black lambs (Chen et al. 2021). Likewise, a slight decline in the ruminal NH_3_-N concentration was observed when Farafra sheep were fed a diet comprising* Lactobacillus acidophilus* (Hamdon et al. 2022). As well, when supplemented the newborn Holstein calves with probiotics, NH_3_-N levels were similar (*P > *0.05) among all the tested animal groups (Wang et al. 2022a).

The reduction of ruminal NH3-N concentrations upon dietary inclusion of *S. cerevisiae* is largely due to its influence on rumen microbial activity and nitrogen utilization. Specifically, *S. cerevisiae* stimulates rumen microbial growth, especially bacteria, leading to increased ammonia incorporation into microbial protein (Sheikh et al. 2022). It also promotes a more efficient rumen microbial population, enhancing the conversion of dietary nitrogen into microbial protein, thus reducing ammonia production (Mousa et al.(2012). Furthermore, *S. cerevisiae* stabilizes rumen pH, creating an optimal environment for microbial growth and protein synthesis, which results in lower ammonia concentrations.

### Ruminal pH value

Ruminal pH is a useful indicator of rumen health following feeding. While thresholds exist to characterize ruminal acidosis, the specific pH value at which damage occurs to the ruminal epithelium, microbial community structure and activity are altered, and feed intake is depressed likely varies among individual animals. The impact of dietary probiotic *Saccharomyces cerevisiae* on some rumen measurements was determined (Mousa et al. 2012). Authors found that the ruminal pH was decreased (*P < *0.05) after three hours of feeding. In addition, including* Bacillus licheniformis* and *Lactobacillus plantarum* into lambs’ diet resulted in a slight significant (*P = *0.002) reduction in the ruminal pH value (Chen et al. 2021). Authors suggested that probiotics encouraged the production of more lactic acid, which led to a reduction in the ruminal pH. Moreover, *Bacillus licheniformis* supplementation caused a lower (*P < *0.05) pH value in the rumen fluid of Chinese Holstein cows (Qiao et al. 2010).

In contrast, the dietary probiotic containing *Bacillus subtilis* led to a slightly significant rise (*P* ≤ 0.05) in the pH value related to the control group (Mousa and Marwan 2019). Also, the ruminal pH value was significantly (*P < *0.01) elevated when Corriedale sheep diet was augmented with *Lactobacillus acidophilus* plus *S. cerevisiae* (Sheikh et al. 2022).

Meanwhile, the ruminal pH value did not differ with the dietary *Saccharomyces cerevisiae* alone or a mixture of *Saccharomyces cerevisiae* and *Bacillus licheniformis* in fattening lambs (Jia et al. 2018). In a study involving male Arabian lambs, Direkvandi et al. (2020b) assessed three probiotic combinations comprised of *Lactobacillus fermentum*, *Lactobacillus plantarum*, *Saccharomyces cerevisiae*, and *Megasphaera elsdenii.* Authors concluded that the mean values of ruminal pH were not impacted by the probiotic supplements. Similarly, the bacterial probiotic supplements had no effect on the ruminal pH value in Farafra sheep (Hamdon et al. 2022). Besides, when supported the Holstein calves’ diets with the probiotics, the ruminal pH value was not changed (*P > *0.05) compared to the control group (Wang et al. 2022a). Low ruminal pH is often associated with acidosis. This condition typically arises when animals consume excessive amounts of readily fermentable carbohydrates, like grains, coupled with insufficient fiber intake. Combining yeast with lactic acid bacteria (LAB) strains can create synergistic effects (McAllister et al. 2011), while the dietary administration of *Saccharomyces cerevisiae* has been shown to help maintain a stable rumen pH (Khan et al. 2016; Amin and Mao 2021). The yeast competes with lactate-producing and promoting lactate-utilizing bacteria, decreasing lactate accumulation (Ogbuewu and Mbajiorgu 2023). In addition, rumen protozoa can control rumen fermentation by slowing down the production of acids that lower the rumen pH (Vibhute et al. 2011).

### Ruminal protozoa count

*Saccharomyces cerevisiae* elevated the ruminal protozoa count significantly (*P < *0.01) by 31.35% in Murrah buffalo bulls at 4 h post-feeding (Kumar et al. 2013). Similarly, the rumen protozoa count in the Corriedale sheep was significantly (*P < *0.01) increased with the dietary probiotics (*Saccharomyces cerevisiae* plus *Lactobacillus acidophilus*) than the control group (Sheikh et al. 2022). In contrast, the dietary probiotic containing *Bacillus subtilis* resulted in a significant decline in total protozoa count (*P* ≤ 0.05) compared to the control group (Mousa and Marwan 2019).

On the other hand, the impact of 4 probiotic formulations (*Lactobacillus fermentum* + *Lactobacillus plantarum*; *Saccharomyces cerevisiae* + *Lactobacillus fermentum* + *Lactobacillus plantarum*; and *Megasphaera elsdenii* + *Saccharomyces cerevisiae* + *Lactobacillus fermentum* + *Lactobacillus plantarum*) on ruminal fermentation in Arabian fattening lambs were assessed (Direkvandi et al. 2020a). Authors observed that probiotic additives had an insignificant influence on the ruminal protozoa population (*P = *0.78). Supplementation of Farafra sheep with *Lactobacillus acidophilus*, *Bacillus subtilis*, or *Ruminococcus albus* did not significantly alter protozoal counts (Hamdon et al. 2022). Discrepancies in reports regarding probiotic impacts on rumen protozoal counts are attributable to many factors involving probiotic strain specificity, variations in experimental design, rumen environment variability, and protozoal species variability. The diverse protozoal population in the rumen, with varying sensitivities to probiotics, can yield inconsistent results depending on the dominant species. Additionally, probiotics can indirectly affect protozoal counts by modifying rumen bacterial populations, which serve as a food source for protozoa, leading to conflicting findings.

### Ruminal microbial crude protein

The ruminal synthesis of microbial crude protein (MCP) was significantly (*P < *0.05) improved with incorporating a multistrain probiotic (including *Propionibacterium freudenreichii, Lactobacillus acidophilus, Lactobacillus casei, Enterococcus faecium, Lactobacillus lactis, Pediococcuscerevisiae, Megasphaeraelsdenii, Bacillus licheniformis*, and one fungus; *Aspergillus oryzae*) in Barki rams’ diets (Soliman et al. 2016). Similarly, mixing *Bacillus licheniformis* plus *Saccharomyces cerevisiae* in the diets of fattening lambs led to a significant enhancement (*P < *0.05) of the ruminal MCP production (Jia et al. 2018). Additionally, the effect of three probiotic combinations [(1) *Lactobacillus fermentum* + *Lactobacillus plantarum*; (2) *Saccharomyces cerevisiae* + *Lactobacillus fermentum* + *Lactobacillus plantarum*; and (3) *Megasphaera elsdenii* + *Saccharomyces cerevisiae* + *Lactobacillus fermentum* + *Lactobacillus plantarum*] were tested on ruminal MCP in the Arabi growing lambs (Direkvandi et al. 2020a). Authors detected that the probiotic supplements improved the ruminal MCP (*P = *0.01). The best MCP value was observed with the combination number 3, but there was no significant difference between 2 and 3. Chen et al. (2021) evaluated the effects of a probiotic mixture, comprising *Bacillus licheniformis*, *Lactobacillus plantarum*, and *Bacillus subtilis*, in lambs. Authors found a sizable (*P < *0.001) upgrading (157%) in the production of the ruminal MCP compared to the control. Moreover, the dietary *Bacillus licheniformis* resulted in a significant rise in the flow of bacterial protein into the duodenum in Holstein cows (Qiao et al. 2010). In conclusion, the positive effects of multi-species probiotic combinations on ruminal microbial crude protein (MCP) synthesis may be attributed to enhanced fiber-degrading bacterial populations and improved nutrient digestibility (Kulkarni et al. 2022).

### Blood constituents

#### Hematological parameters

Blood hematology is an important indicator of an animal's physiological and health status, including its exposure to infections. Therefore, assessing blood indices is crucial in livestock farms for monitoring animal health. Table 5 summarizes the effects of probiotics on hematological and biochemical parameters in sheep. In lactating Kamieniec ewes, supplementation with 30 g/day of *Saccharomyces cerevisiae* dried yeast increased white blood cell (WBC) counts, red blood cell (RBC) counts, hematocrit (HCT), and hemoglobin (Hb) concentration (Milewski and Sobiech 2009). Similarly, Najdi male lambs receiving a probiotic containing *Lactobacillus sporogenes* and *Saccharomyces cerevisiae* showed significant increases (*P < *0.05) in Hb, packed cell volume (PCV), RBC counts, and WBC counts compared to controls (Hussein 2014). El-Mehanna et al. (2017), found that feeding Noemi male lambs probiotics containing *Lactobacillus bulgaricus* recorded that the total WBCs count was raised (*P < *0.05), while the supplementary probiotic did not affect RBCs count, Hb level, and PCV values. Oral supplementation with *Lactobacillus bulgaricus* for 15 days significantly increased *(P = *0.001) total WBC and lymphocyte counts in Ossimi male lambs (El-Ashker et al. 2018). The counts of total WBCs and lymphocytes were increased (*P < *0.05) after 30 days of receiving the Barki lambs with a dietary probiotic involving *Bacillus subtilis* at 8.4 × 10^4^ CFU/day (Mousa et al. 2019). Moreover, the impact of bacterial probiotics (*Bacillus subtilis*; *Lactobacillus acidophilus*) involved in commercial preparations were assessed on blood haematology in the Farafra lambs fed a control palm leaf hay-based diet (Hamdon et al. 2022). The tested probiotics had no effect on HCT, WBC count, MCV, or MCHC, but significantly increased hemoglobin concentration and RBC count (*P = *0.001) in the Farafra lambs as shown in previous work.

**Table 5:** The effects of probiotics on hematological and biochemical parameters in sheep

Item	Type of probiotics	Dose/Level	Sheep breed	Main findings	Reference
Hematological Parameters	*Saccharomyces cerevisiae* (dried yeast)	30 g/day	Kamieniec lactating ewes	*Saccharomyces cerevisiae* administration resulted in significant elevations of WBC, RBC, HCT, and Hb levels	Milewski and Sobiech (2009)
	*Lactobacillus sporogenes*, *Saccharomyces cerevisiae*	5–10 g/kg probiotic *Lactobacillus sporogenes(*7.500 × 10^6^ cfu), *Saccharomyces cerevisiae* (125.000 × 10^6^ cfu)	Najdi male lambs	Najdi male lambs fed diets supplemented with *Lactobacillus sporogenes* and *Saccharomyces cerevisiae* (5–10 g/kg) exhibited significantly higher (*P < *0.05) Hb, PCV, RBC, and WBC levels compared to the control diet	Hussein (2014)
	*Lactobacillus bulgaricus*	Animals were orally given 50 ml of probiotic fermented cow's milk enriched with lactic acid bacteria (1 × 10^10^ CFU/g)	Noemi male lambs	Supplementation with* Lactobacillus bulgaricus* resulted in a significant increase in WBC counts (*P < *0.05), while RBC counts, Hb levels, and PCV remained unchanged	El-Mehanna et al. (2017)
	*Lactobacillus bulgaricus*	A dose rate of 0.5 g/head as an oral drench twice daily for 30 consecutive days	Ossimi male lambs	A statistically significant increase in white blood cell (WBC) and lymphocyte counts was observed (*P = *0.001)	El-Ashker et al. (2018)
	*Bacillus subtilis*	8.4 × 10^4^ CFU/day for 30 days	Barki lambs	White blood cell (WBC) and lymphocyte counts were significantly elevated (*P < *0.05)	Mousa et al. (2019)
	*Bacillus subtilis*, *Lactobacillus acidophilus* (commercial mix)	4 g (BAC: 2 × 10^11^ CFU/g, Laco: 1 × 10^12^ CFU/g)	Farafra growing lambs	A significant increase in Hb and RBC counts was observed (*P = *0.001); however, HCT, WBC counts, MCV, and MCHC showed no significant changes	Hamdon et al. (2022)
	*Lactobacillus acidophilus, L. bulgaricus, Bacillus licheniformis, Bifidobacterium bifidum*	2 × 10⁹ or 4 × 10⁹ CFU/g ± 2 × 10⁷ CFU/g dry yeast	Male Saidi sheep	Supplementation with probiotic and yeast resulted in a significant increase in WBC counts (*P = *0.002), but no other parameters were significantly affected	Saleem et al. (2024)
Biochemical parameters	*Saccharomyces cerevisiae* (dried yeast)	30 g/day	Kamieniec lactating ewes	In lactating ewes, the addition of *Saccharomyces cerevisiae* resulted in a significant increase in glucose levels and a significant decrease in creatinine levels	Milewski and Sobiech (2009)
	*Pediococcus acidilactici* (10⁶ CFU/g), *Pediococcus pentosaceus* (1.3 × 10⁶ CFU/g)	0.5 or 1 g/day	Weaned Saidi lambs	The treatment resulted in no significant changes in glucose, total protein, albumin, or globulin concentrations. Conversely, urea and cholesterol concentrations were significantly reduced (*P < *0.05)	Saleem et al. (2017)
	*Lactobacillus acidophilus* (2.5 × 10⁷ CFU/g), *L. casei* (2.5 × 10⁷ CFU/g), *Bifidobacterium thermophilum* (2.5 × 10⁷ CFU/g), *Enterococcus faecium* (2.5 × 10⁷ CFU/g)	2 g/day	Lactating Sanjabi ewes	The probiotics administration indicated no significant effect on glucose, cholesterol, HDL, LDL, triglycerides, ALT, ALP, LDH	Kafilzadeh et al. (2019)
	*Bacillus subtilis*	8.4 × 10^4^ CFU/day for 30 days	Weaned Barki lambs	In weaned Barki lambs fed diets containing *Bacillus subtilis*, no significant changes were observed in total protein, albumin, ALT, AST, urea, or creatinine concentrations	Mousa et al. (2019)
	*Bacillus subtilis*, *Bacillus licheniformis* (5 × 10⁹ CFU)	1 g/day (lambs), 1 or 3 g/day (sheep)	Growing lambs and adult sheep	Sheep fed diets supplemented with 1 or 3 g/day of *Bacillus subtilis* and *Bacillus licheniformis* showed a significant increase in total protein, a significant increase in albumin (specifically at the 3 g/day dose), a significant reduction in cholesterol, and no significant alterations in creatinine, ALT, or AST levels	Devyatkin et al. (2021)
	*Bacillus subtilis* (2 × 10^11^ CFU), *Lactobacillus acidophilus* (10^12^ CFU), *Ruminococcus albus* (6 × 10⁸ CFU)	4 g/day	Farafra lambs	The administration of *Bacillus subtilis* and *Ruminococcus albus* in the diet did not significantly alter glucose, triglyceride, AST, or ALT concentrations. Conversely, it led to a significant increase in albumin (*P < *0.001) and total protein (*P = *0.001), and a significant decrease in globulin, urea nitrogen, creatinine (*P = *0.024), and cholesterol (*P = *0.031)	Hamdon et al. (2022)

In a separate study by Saleem et al. (2024), the effects of supplementing the basal diet (50:50 roughage/concentrate) of male Saidi sheep (average body weight 54.14 ± 1.67 kg) for 105 days with a multi-strain probiotic blend (*Lactobacillus acidophilus*, *L. bulgaricus*, *Bacillus licheniformis*, and *Bifidobacterium bifidum*) at levels of 2 × 10⁹ or 4 × 10⁹ CFU/g, with or without 2 × 10⁷ CFU/g of dry yeast, were investigated. The authors reported no significant effects of the probiotic blends on hematological parameters, except for a significant increase in WBC count (*P = *0.002) observed with both doses of the multi-strain probiotic blend plus dry yeast, compared to the multi-strain probiotic blend alone and the control group.

In a study involving suckling Holstein calves, Al-Saiady (2010) found that probiotic supplementation with a combination of *Lactobacillus acidophilus* and *Lactobacillus plantarum* resulted in a significant increase in WBC counts, while PCV and Hb concentrations remained unchanged relative to the control group. Riddell et al. (2010) demonstrated that dietary administration of *Bacillus subtilis* and *Bacillus licheniformis* had no significant impact on HCT values in calves. A similar trend was detected with determined the impact of commercial probiotics (containing strains of *Bacillus, Pediococcus, Lactococcus, Lactobacilli, Bifidobacteria,* and *Saccharomyces*; 12 g/day) on blood parameters of neonatal female Bulgarian calves (Dimova et al. 2013).

As direct studies on the influence of probiotics on sheep hematology are limited, we have included supporting data from calves and other ruminants, acknowledging the physiological similarities within these species. A study reported no significant alterations in Hb concentration, RBC, or WBC counts in female Holstein calves. While the authors asserted improved health in probiotic-fed calves compared to controls, this claim lacks substantiation, as no supporting data regarding disease incidence, growth rate, or other relevant health parameters were presented. Similarly, Agazzi et al. (2014) supplemented female Holstein calves with a probiotic combination (1 g/calf/day; 1.8 × 10^10^ CFU/g) containing *Lactobacillus animalis*, *Lactobacillus paracasei*, and *Bacillus coagulans*. Their results also showed no significant differences in blood count values among the treated groups. Growing heifers were supplemented with graded levels (0, 10, and 20 g) of a probiotic blend (*Bacillus subtilis* plus *Bacillus licheniformis*; 1:1; 2 × 10⁹ CFU of each) via daily oral administration (Shetawy et al. 2022). Authors observed that the probiotics supplements did not significantly affect the Hb content, the counts of RBCs, WBCs, and platelets (PLT), lymphocyte, granulocyte, in blood. The observed impacts of probiotics on blood hematology are conflicting. This is likely due to several factors related to both probiotic supplementation (dose, timing, and species of probiotic) and the animals themselves (age, physiological status, and gender).

#### Biochemical parameters

Blood biochemistry reflects nutrient status post-absorption and metabolic processes (Herdt et al. 2000). Assessing changes in blood biochemical parameters can provide valuable insights into animal health and physiological responses to new feedstuffs or exposure to abiotic or biotic stressors (Sheiha et al. 2020; Taies and Al-Samarai 2024).

In sheep, the dietary probiotic (*Saccharomyces cerevisiae*) supplements elevated the glucose content while reducing the creatinine content in the blood plasma of ewes compared with the control group (Milewski and Sobiech 2009). As noted earlier, Saccharomyces cerevisiae-mediated enhancement of carbohydrate fermentation increases VFA production, subsequently contributing to hepatic glucose synthesis. Supplementation of weaned Saidi lambs with two doses (0.5 or 1 g/day) of a bacterial probiotic blend (*Pediococcus acidilactici* at 10⁶ CFU/g and *Pediococcus pentosaceus* at 1.3 × 10⁶ CFU/g) had no significant effect on serum glucose, total protein, albumin, or globulin concentrations (Saleem et al. 2017). However, serum urea and cholesterol levels were significantly reduced. The higher probiotic dose (1 g/day) significantly decreased cholesterol concentration compared to the lower dose (0.5 g/day).

The effects of a dietary probiotic combination (2 g/day) (comprising* Lactobacillus acidophilus*; 2.5 × 10^7^ CFU/g, *Lactobacillus casei*; 2.5 × 10^7^ CFU/g,* Bifidobacterium thermophilum*; 2.5 × 10^7^ CFU/g; *Enterococcus faecium*, 2.5 × 10^7^ CFU/g) were studied on some blood biochemicals of the lactating Sanjabi ewes (Kafilzadeh et al. 2019). Authors documented that the probiotic combination has no effects (*P > *0.05) on concentrations of glucose, cholesterol, high-density lipoprotein (HDL), low-density lipoprotein (LDL), triglycerides, and alanine aminotransferase (ALT), alkaline phosphatase (ALP), and lactate dehydrogenase activities. Mousa et al. (2019) supplemented weaned Barki lambs with dietary probiotics involving *Bacillus subtilis* (8.4 × 10^4^ CFU/day) for 30 days in comparison to the control, the probiotic had no significant impacts on serum total protein, albumin, ALT, AST, urea, and creatinine values.

The impact of dietary supplementation with *Bacillus subtilis* and *Bacillus licheniformis* probiotics (5 × 10⁹ CFU) at 1 g/day for growing lambs and 1 or 3 g/day for adult sheep on biochemical parameters in sheep was determined by Devyatkin et al. (2021). After 30 days of addition, authors recorded that the concentration of the measured blood metabolites in all the tested groups was within acceptable physiological range. In sheep, the serum total protein and albumin concentrations were increased with both tested doses of the probiotic, while albumin concentration was elevated with the higher dose (3 g/day) only. Also, globulin content increased by 10.8% attributable to probiotics supplementation. The probiotic supplements had no considerable influence on concentrations of creatinine and cholesterol, and activities of ALT and AST in sheep. There was a tendency to reduce the concentration of serum cholesterol.

A study by Hamdon et al. (2022) assessed the effects of three different dietary probiotics (4 g/day each) on the blood biochemical parameters of Farafra lambs fed a palm leaf hay-based diet. The probiotics contained *Bacillus subtilis* (2 × 10^11^ CFU), *Lactobacillus acidophilus* (10^12^ CFU), or *Ruminococcus albus* (6 × 10⁸ CFU). The authors reported no significant effects of any of the probiotics on blood glucose or triglyceride concentrations, AST or ALT activities. However, all tested probiotics significantly increased albumin concentrations and decreased globulin and urea-nitrogen concentrations. Compared to the control group, *B. subtilis* and *R. albus* both significantly increased total protein concentrations (*P = *0.001) and decreased creatinine (*P = *0.024) and cholesterol (*P = *0.031) concentrations in blood serum.

A study by Mousa and Marwan (2019) investigated the effects of a dietary probiotic containing *Bacillus subtilis* (0.0252 × 10⁶ CFU/kg diet/day) on the blood biochemistry of buffalo calves. Authors reported that the probiotic group exhibited significant increases (*P* ≤ 0.05) in total serum proteins, albumin, triglycerides, AST, and ALT concentrations. Conversely, serum urea and creatinine concentrations significantly decreased, while globulin concentrations remained unchanged compared to the control group. In another research, Wang et al. (2022b) evaluated the effects of two doses (low; 0.12 g/day and high; 1.2 g/day) of a probiotic blend on the blood parameters of Holstein calves. The blend contained *Lactobacillus plantarum* (10⁸ CFU/g), *Pediococcus acidilactici* (10⁸ CFU/g), *Pediococcus pentosaceus* (10⁸ CFU/g), and *Bacillus subtilis* (10⁷ CFU/g). The previous study also indicated an increase in serum total protein concentrations with the high probiotic dose, but no effect on glucose concentrations.

### Oxidative biomarkers

Oxidative biomarkers are commonly used to indicate an animal’s physiological and health status (Ranade et al. 2014; Al-Janabi and Al-Samarai 2023). The cells are protected by antioxidants and intracellular enzymes such as superoxide dismutase (SOD), glutathione peroxidase (GSH-Px), and catalase, which eliminates peroxides and superoxides to prevent the formation of more reactive compounds by the reaction with metal catalysts (Miller et al. 1993; Ibrahim et al. 2024). Probiotics from lactic acid-producing bacteria exhibit high antioxidant activity (Tang et al. 2018), which may modulate the antioxidant activity of the host.

The impact of *Bacillus subtilis* supplementation (8.4 × 10^4^ CFU/day) in Barki lambs were examined on anti-oxidative stress properties (Mousa et al. 2019). After 30 days of probiotic treatment, authors indicated that the *Bacillus subtilis* resulted in a remarkable decrease in the malondialdehyde values while increasing the total antioxidant capacity (TAC) and total reduced glutathione enzymes in blood serum relative to control. As well, two strains of *Bacillus subtilis* (BS1 and BS2) reduced the serum MDA, and elevated the TAC and GSH-PX levels in mice (Li et al. 2019). A study by Izuddin et al. (2020) explored the effects of dietary postbiotics (*Lactobacillus plantarum* RG14, RG11, and TL1) on serum antioxidant activity in weaned Dorper lambs. Authors reported that the *L. plantarum* RG14 postbiotic reduced serum malondialdehyde (MDA) concentrations and increased GSH-Px activity compared to the control group.

Furthermore, the bacterial probiotics combination containing *Lactobacillus acidophilus, L. bulgaricus, Bacillus licheniformis,* and *Bifidobacterium bifidum* at a level of (2 × 10^9^ cfu/g) or (4 × 10^9^ cfu/g) remarkably enhanced the serum antioxidant parameters in the tested sheep fed the experimental diets (Saleem et al. 2024). Specifically, The probiotic supplements led to statistically significant increases in GSH-Px and TAC values, while mean MDA values were significantly reduced compared to the control group (Saleem et al. 2024).

At the same time, the effect of *Bacillus licheniformis* (4 × 10^9^ CFU) and *Saccharomyces cerevisiae* (3.2 × 10^9^ CFU) and a mixture of them (*Bacillus licheniformis*; 6 × 10^9^ CFU and *Saccharomyces cerevisiae*; 4 × 10^9^ CFU) were tested on oxidative biomarkers in fattening lambs (Jia et al. 2018). Authors detected that all probiotic treatments did not have any considerable effect on the serum levels of MDA and total TAC. However, the activity of SOD and GSH-Px was significantly higher in the mixture group than that in the control. Also, Wang et al. (2022a) supplemented Holstein calves with 1.2 g/day of a probiotic blend (consisting of 10^8^ CFU/g of *Lactobacillus plantarum*, 10^8^ CFU/g of *Pediococcus acidilactici*, 10^8^ CFU/g of *Pediococcus pentosaceus*, 10^7^ CFU/g of and *Bacillus subtilis*). Authors found that the probiotic decreased the MDA while increasing the TAC and the activity of SOD compared to the control.

### Impacts on immune function

Probiotics can stimulate the production of antibodies, enhancing both humoral and cell-mediated immunity. This can lead to improved resistance against infections. Incorporating probiotics into the diet of sheep can significantly bolster their immune function, leading to better health outcomes and improved productivity. The total serum protein, which includes both non-immunoglobulin and immunoglobulin proteins, is associated with the animal's immune system (Wang et al. 2020). A considerable increase was detected in the serum lysozyme activity after 30 days of supplementing the Barki lambs with *Bacillus subtilis* with 8.4 × 10^4^ CFU (Mousa et al. 2019). After 80 days of the oral administration the Holstein calves with a probiotic blend (involving *Bacillus subtilis*), authors found an increase in the serum total protein and the immunoglobulin with the higher dose of probiotics (1.2 g/day) (Wang et al. 2022a).

The probiotic (*Bacillus subtilis, Bacillus licheniformis,* and *Lactobacillus plantarum*) treatment significantly (*P = *0.001) increased the concentration of IgG in lambs (Chen et al. 2021). Moreover probiotic supplementation (*Bacillus licheniformis*; 6 × 10^9^ CFU and *Saccharomyces cerevisiae*; 4 × 10^9^ CFU) advanced the IgA, IgM, and IgG levels (by 39.53, 68.75, and 52.69%, respectively) compared to the control in the fattening lamb’s serum (Jia et al. 2018). As shown above, Saleem et al. (2024) investigated the effects of supplementing the basal diet (50:50 roughage/concentrate) of male Saidi sheep (average body weight 54.14 ± 1.67 kg) with a multi-strain probiotic blend (*Lactobacillus acidophilus*, *L. bulgaricus*, *Bacillus licheniformis*, and *Bifidobacterium bifidum*) at levels of 2 × 10⁹ or 4 × 10⁹ CFU/g, with or without *Saccharomyces cerevisiae* (2 × 10⁷ CFU/g). Authors observed a slight increase in serum IgM levels with the inclusion of the multi-strain probiotic blend. Additionally, the supplemented diets led to significant increases in serum IgG and IgA concentrations, and lysozyme activity compared to the control. The gut lining serves as a protective barrier, preventing harmful substances from entering the bloodstream. Probiotics help strengthen this barrier by promoting the production of mucin and tight junction proteins, reducing the risk of"leaky gut"and related immune issues.

Probiotics enhance the production of animal IgG and IgM antibodies by modulating the gut-associated lymphoid tissue (GALT). They activate immune cells in the GALT, stimulating the proliferation of B lymphocytes and antibody production, and influence cytokine release to promote Th1 and Th2 immune responses. Additionally, probiotics directly stimulate B lymphocytes, enhancing proliferation, plasma cell differentiation, and overall antibody response. They also improve gut barrier function by increasing mucin, tight junction proteins, and antimicrobial peptides, thereby regulating immune responses and preventing chronic inflammation. Furthermore, probiotics modulate Toll-like receptor (TLR) signaling, impacting cytokine production and B cell activation, and enhance the function of antigen-presenting cells, which are crucial for guiding B cell antibody production.

## Conclusion

The current review comprehensively highlights the beneficial impacts of probiotic administration on sheep production, promoting health, sustainability, and environmental benefits. Dietary probiotics administration can modulate the rumen microbiota, leading to enhanced nutrient digestibility, improved blood parameters, and boosted immune function, ultimately increasing overall productivity. Additionally, probiotics can reduce methanogenic bacteria and decrease methane emissions on sheep farms. Recommendations for probiotic supplementation in sheep diets vary depending on diet composition, the animal's physiological status, and the specific probiotic used. Further research, particularly in vivo, is needed to elucidate the mechanisms by which probiotics mitigate methane emissions, confirming the results observed in in vitro trials. Moreover, encapsulating multi-strain probiotics may enhance their efficacy in modulating the rumen microbiota and thus improve sheep performance. Further studies are needed to explore the use of multi-strain probiotics or to test new types of probiotics to enhance production and reduce methane emissions.

## Data Availability

All data are available within the manuscript.
